# Risk of neurodevelopmental impairment in Swedish preterm children treated for necrotizing enterocolitis: retrospective cohort study

**DOI:** 10.1093/bjsopen/zrae131

**Published:** 2024-11-08

**Authors:** Nele Brusselaers, Johanna Simin, Helene E. Lilja

**Affiliations:** Department of Women’s and Children’s Health, Karolinska Institutet, Stockholm, Sweden; Global Health Institute, Department of Family Medicine and Population Health, University of Antwerp, Antwerp, Belgium; Department of Public Health and Primary Care, Ghent University, Ghent, Belgium; Global Health Institute, Department of Family Medicine and Population Health, University of Antwerp, Antwerp, Belgium; Department of Women’s and Children’s Health, Karolinska Institutet, Stockholm, Sweden; Department of Paediatric Surgery, Karolinska University Hospital, Stockholm, Sweden

## Abstract

**Background:**

As the survival of preterm infants has increased, the management of long-term complications, especially neurodevelopmental impairment, becomes increasingly important. The aim of this study was to investigate the risk of neurodevelopmental disorders in preterm babies receiving medical or surgical treatment for necrotizing enterocolitis, compared with other preterm babies and preterm babies who received abdominal surgery for other indications.

**Methods:**

In this nationwide Swedish cohort study, including all liveborn preterm babies born between 1998 and 2019, the risk of attention deficit (and hyperactivity) disorder, autism spectrum disorders, cerebral palsy and intellectual disability was assessed by multivariable Cox regression, expressed as hazard ratios and 95% confidence intervals (c.i.).

**Results:**

Of the surgically (*n* = 384) and medically (*n* = 709) treated preterm infants with necrotizing enterocolitis, neurodevelopmental disorders were present in 32% (HR 2.24, 95% c.i. 1.86 to 2.69) and 22% respectively (HR 1.40, 95% c.i. 1.19 to 1.65), compared with 21% (HR 1.63, 95% c.i. 1.40 to 1.91) in the abdominal surgery group (*n* = 844) and 13% (reference) among other preterm infants (*n* = 78 972). The highest relative increases were for intellectual disability (HR 3.60, 95% c.i. 2.65 to 4.89) in the surgical necrotizing enterocolitis group and abdominal surgery group (HR 2.84, 95% c.i. 2.12 to 3.80) compared with the control preterm group, and for cerebral palsy (respectively HR 2.74, 95% c.i. 2.04 to 3.68 and HR 2.54, 95% c.i. 1.87 to 3.44). Medically treated necrotizing enterocolitis was associated with autism (HR 1.67, 95% c.i. 1.34 to 2.08), without significant increases for the other specific outcomes. Both surgically treated groups were also strongly associated with both attention deficit (and hyperactivity) disorder and autism.

**Conclusion:**

Surgically treated necrotizing enterocolitis, medically treated necrotizing enterocolitis and abdominal surgery for other indications in preterm infants were all associated with an increased risk of impaired neurodevelopmental outcomes, compared with other preterm infants.

## Teaser

Surgically and medically treated necrotizing enterocolitis were both associated with an increased risk of neurodevelopmental disorders, as well as abdominal surgery for other indications, suggesting that per-operative conditions may also have an effect on top of the systemic inflammation related to necrotizing enterocolitis.

## Introduction

Necrotizing enterocolitis (NEC) is the most severe gastrointestinal disorder, affecting 3–17% of extremely preterm or very low-birthweight infants (pooled prevalence 6–7%)^1^, and is responsible for approximately one-tenth of all neonatal deaths^2^. One of four infants who develop NEC die, and 50% of extremely low-birthweight infants need surgical treatment for NEC^2^.

Although it is unclear why some preterm babies develop NEC, it seems that low gestational age, low birthweight, an underdeveloped gastrointestinal barrier function and the early gastrointestinal microbiome play a central role^3–6^. NEC is characterized by inflammation of the bowel, which can progress to bowel necrosis and bowel perforation^7^. The inflammatory process in the bowel leads to systemic inflammation, which may also affect distant organs such as the brain^7,8^. Initial treatment of NEC is conservative (medical), with bowel rest and broad-spectrum antibiotics^7^. However, up to 50% need emergency surgery with resection of the necrotic bowel due to clinical progression of the disease^7^. Surgery in preterm infants coincides with a time interval of rapid brain development including neurogenesis, neuronal migration, maturation, apoptosis and synaptogenesis^7^. The brain of the infant is more vulnerable to factors that interrupt these processes such as inflammation and general anaesthetic agents^7,9,10^. Preterm babies are at increased risk of neurodevelopmental problems, especially extremely preterm babies and those who develop complications such as retinopathy of prematurity (ROP), brain injury, bronchopulmonary dysplasia or sepsis^11–13^. Neurodevelopmental impairments include a broad group of disabilities involving some form of disrupted brain development, including attention deficit and hyperactivity disorder (ADHD)/attention deficit disorder (ADD), autism spectrum disorders and intellectual disability. A meta-analysis on preterm babies with NEC showed that neurodevelopmental impairment was present in 40% of the infants, with cerebral palsy being most frequently diagnosed^14^. The surgically treated group was at highest risk^14^. They also assessed that the risk of neurodevelopmental impairment was 40% higher than age-matched preterm babies without NEC, for blindness, hearing problems and ADHD/ADD^14^.

The aim of this study was to investigate the risk of neurodevelopmental disorders in preterm babies receiving medical or surgical treatment for NEC, in comparison to other preterm babies and preterm babies who received abdominal surgery for other indications.

## Methods

**Fig. 1:**
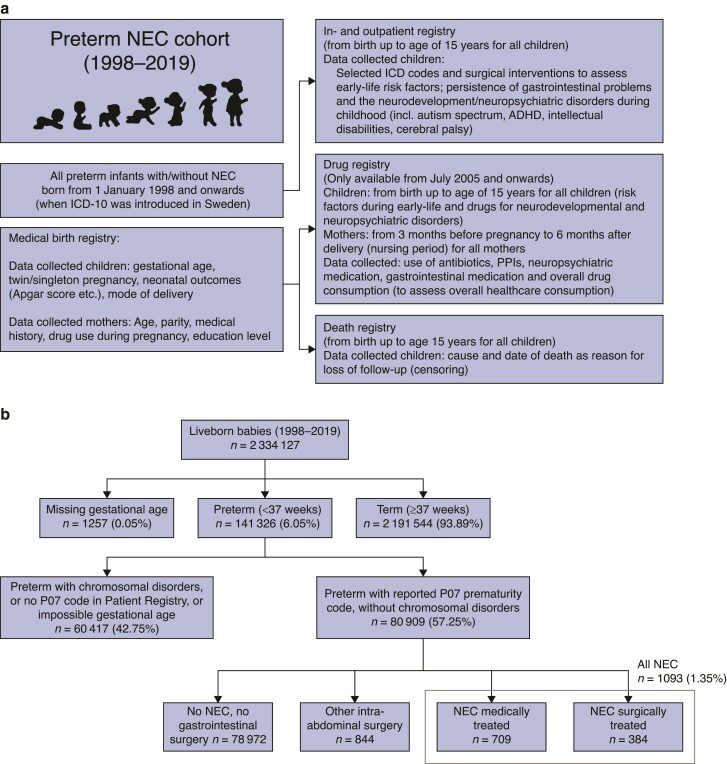
a Registry linkage and b selection of the study cohort (flow chart) ICD, international classification of diseases; NEC, necrotizing enterocolitis; ADHD, attention deficit and hyperactivity disorder; PPI, proton pump inhibitor.

This nationwide study includes all liveborn preterm babies born between 1 January 1998 and 31 December 2019. Preterm birth was defined as pregnancies resulting in liveborn babies with a gestational age below 37 completed weeks, as defined by the World Health Organization^15^. Data were collected from the following nationwide health registries, for which data registration is compulsory resulting in high completeness and validity: the Medical Birth Registry (established in 1973, mothers/children)^16^, the outpatient Prescribed Drug Registry (children)^17^, the Inpatient and specialist Outpatient care Registries (nationwide complete since 1987 and 2001 respectively, children)^18^, the Cause of Death Registry (established in 1952, children)^19^ (*Fig. 1a* and *Table S1*). Babies born with diagnosed chromosomal disorders (including Down and other syndromes) were excluded, as well as children without the ICD-10 code P07 for preterm birth, and those with impossible gestational age (*Fig. 1b*). The study was approved by the Swedish National Ethics Committee (2020-05027).

### Exposure

All preterm infants were divided into four mutually exclusive subgroups: medically treated NEC defined by ICD-10 code P77; surgically treated NEC defined by ICD-10 code P77 in combination with surgical procedure code JFB (resection of small bowel or colon) and/or JFF (intestinal exteriorization and creation of intestinal stoma); preterm infants without P77 code receiving abdominal surgery in the neonatal interval (before 44 weeks of gestation) for other indications defined by selected abdominal surgical procedure codes; and all preterm infants not falling under the other three groups (*Table S1*). Infants with spontaneous intestinal perforation (ICD-10 code P78.0) were not included.

### Outcome

ADHD/ADD, autism spectrum disorders, cerebral palsy and intellectual disability were defined by ICD-10 codes (see *Table S1*) as registered in the Patient Registries.

### Covariates

Regarding the child and pregnancy, data were collected from the Medical Birth Registry on gestational age (categorized as extremely preterm (22–27 weeks), early preterm (28–32 weeks) and late preterm (33–36 weeks)); sex of the child (categorized as boys or girls); mode of delivery (vaginal, elective caesarian section, acute caesarian section); birth weight (small, normal or large for gestational age); Apgar score at 5 min to assess vitality, which ranges between 0 and 10 (categorized as low Apgar (<7) compared with ≥7); and parity (categorized as <3 and ≥3). From the drug registry, information was collected on prescribed and dispensed use (yes or no) of potential microbiome-modulating drugs administered during the first year of life^20–23^. Drugs such as proton pump inhibitors (PPIs)^24^ and systemic antibiotics^4,25^ have a potential microbiome-disruptive effect, and non-steroidal anti-inflammatory drugs (NSAIDs) a protective, anti-inflammatory effect. This information was only available for those born from July 2005 and onwards (establishment of the Prescribed Drug Registry).

Maternal characteristics were collected from the Medical Birth Registry and included maternal age at the time of delivery (continuous variable), and maternal history of these neurodevelopmental disorders as recorded in the medical history relevant to the pregnancy (yes or no). The date of death was collected for all children from the Cause of Death Registry and used for censoring.

### Statistical methods

The risk of the four outcomes (not mutually exclusive) and a composite outcome combining all outcomes was estimated using a multivariable Cox regression model, expressed as hazard ratios (HR) and 95% confidence intervals and adjusted for all covariates. The preterm group without NEC or surgery was used as a reference. Follow-up started at the time of delivery, and all children were followed up until the occurrence of any of the outcomes, death or the end of the study interval (December 2020), whichever came first. For the subgroup analyses (2005–2019), including paediatric drugs during the first year of life, follow-up started 1 year after birth. For these analyses, everyone with a diagnosis during the first year or too short follow-up were excluded. Covariates with missing estimates for the univariable model were not estimable and were not considered for the multivariable model. As gestational age is a main risk factor for neurodevelopmental disorders, this was also used to stratify our analyses. Complete case analysis was used for all models. All analyses were performed using R version 4.1.1 (R Core Team, Vienna, Austria).

## Results

In total, 80 915 preterm babies were included, of which 1093 (1.35%) developed NEC (*Fig. 1b*). Two-thirds of infants with NEC (*n* = 709, 64.8%) were treated conservatively, and one-third received abdominal NEC surgery (*n* = 384, 35.1%). There were 844 children who received abdominal surgery for other indications. The control group consisted of 78 972 preterm infants without NEC or abdominal surgery.

More children with NEC were born extremely premature (before 28 weeks), with 59.1% and 65.6% of the medically and surgically treated NEC groups, compared with 15.4% of those receiving abdominal surgery for other indications, or 6.8% of the control group (*Table 1*).

**Table 1:** Descriptive characteristics of all four groups of preterm babies, categorized by medically or surgically treated necrotizing enterocolitis, other abdominal surgery and all other preterm babies born in Sweden between 1998 and 2019

	Control	Other intra-abdominal surgery	NEC		Total
Variable			Medically treated NEC	Surgically treated NEC	
	(*n* = 78 972)	(*n* = 844)	(*n* = 709)	(*n* = 384)	(*n* = 80 909)
**Pregnancy characteristics**					
**Gestational age**					
20–27 weeks	5393 (6.8)	130 (15.4)	419 (59.1)	252 (65.6)	6194 (7.7)
28–32 weeks	18 587 (23.5)	164 (19.4)	224 (31.6)	95 (24.7)	19 070 (23.6)
33–36 weeks	54 363 (68.8)	503 (59.6)	58 (8.2)	33 (8.6)	54 957 (67.9)
Missing	629 (0.8)	47 (5.6)	8 (1.1)	4 (1.0)	688 (0.9)
**Sex**					
Male	42 950 (54.4)	472 (55.9)	379 (53.5)	228 (59.4)	44 029 (54.4)
Female	36 022 (45.6)	372 (44.1)	330 (46.5)	156 (40.6)	36 880 (45.6)
**Mode of delivery**					
Vaginal	40 811 (51.7)	362 (42.9)	259 (36.5)	130 (33.9)	41 562 (51.4)
Elective caesarian section	23 397 (29.6)	319 (37.8)	275 (38.8)	165 (43.0)	24 156 (29.9)
Acute caesarian section	14 764 (18.7)	163 (19.3)	175 (24.7)	89 (23.2)	15 191 (18.8)
**Birth weight by gestational age**					
Appropriate	67 983 (86.1)	677 (80.2)	556 (78.4)	296 (77.1)	69 512 (85.9)
Small	8342 (10.6)	129 (15.3)	149 (21.0)	82 (21.4)	8702 (10.8)
Large	2647 (3.4)	38 (4.5)	4 (0.6)	6 (1.6)	2695 (3.3)
**Apgar, 5 min**					
<7	6534 (8.3)	129 (15.3)	229 (32.3)	149 (38.8)	7041 (8.7)
≥7	72 438 (91.7)	715 (84.7)	480 (67.7)	235 (61.2)	73 868 (91.3)
**Parity, number of births**					
<3	62 301 (78.9)	660 (78.2)	574 (81.0)	304 (79.2)	63 839 (78.9)
≥3	16 671 (21.1)	184 (21.8)	135 (19.0)	80 (20.8)	17 070 (21.2)
**Maternal characteristics**					
Age (years), median (i.q.r.)	30 (27–34)	30 (26–34)	31 (27–35)	30 (27–35)	30 (26–35)
Maternal history of outcomes	287 (0.4)	3 (0.4)	5 (0.7)	4 (1.0)	299 (0.4)
**Study interval (date of birth)**					
Jan 1998–Jun 2005	21 064 (26.7)	248 (29.4)	108 (15.2)	53 (13.8)	21 473 (26.5)
Jul 2005–Dec 2019	57 908 (73.3)	596 (70.6)	601 (84.8)	331 (86.2)	59 436 (73.5)
**Prescribed drug use during first year of life (2005–2019)—outpatient use only**					
Non-steroidal anti-inflammatory drugs	235 (0.3)	3 (0.4)	2 (0.3)	0 (0.0)	240 (0.3)
Proton pump inhibitors	1536 (1.9)	123 (14.6)	67 (9.4)	51 (13.3)	1777 (2.2)
Systemic antibiotics	11 629 (14.7)	222 (26.3)	105 (14.8)	59 (15.4)	12 015 (14.9)
**Paediatric outcomes**					
Any neurodevelopmental outcome	9848 (12.5)	180 (21.3)	158 (22.3)	121 (31.5)	10 307 (12.7)
Age (years), median (range)	6.0 (0.0–15.0)	3.7 (0.2–14.7)	3.3 (0.4–14.9)	2.9 (0.2–13.8)	6.0 (0.0–15.0)
ADHD/ADD	6318 (8.0)	111 (13.2)	70 (9.9)	47 (12.2)	6546 (8.1)
Age (years), median (range)	7.8 (0.0–15.0)	6.6 (0.2–14.7)	6.1 (0.4–12.8)	5.0 (0.2–12.7)	7.8 (0.0–15.0)
Autism spectrum	4252 (5.4)	80 (9.5)	89 (12.5)	67 (17.4)	4488 (5.5)
Age (years), median (range)	5.3 (0.0–15.0)	3.8 (0.5–14.0)	3.6 (0.5–14.9)	4.0 (0.5–14.4)	5.3 (0.0–15.0)
Cerebral palsy	1460 (1.8)	45 (5.3)	40 (5.6)	48 (12.5)	1593 (2.0)
Age (years), median (range)	1.9 (0.0–14.9)	1.7 (0.6–12.7)	1.6 (0.7–9.6)	1.7 (0.8–5.7)	1.9 (0.0–14.9)
Intellectual disability	1458 (1.8)	52 (6.2)	31 (4.4)	45 (11.7)	1586 (2.0)
Age (years), median (range)	6.4 (0.1–14.9)	6.1 (0.8–13.8)	5.8 (1.2–14.4)	5.3 (1.1–9.7)	6.4 (0.1–14.9)
Death (first year)	1392 (1.8)	64 (7.6)	142 (20.0)	91 (23.7)	1689 (2.1)

Values are *n* (%) unless otherwise indicated. ADHD/ADD, attention deficit (and hyperactivity) disorder; NEC, necrotizing enterocolitis; i.q.r., interquartile range.

The male predominance was most pronounced in the surgically treated NEC group (59.4%), and lowest in the medically treated NEC group (53.5%).

A larger proportion of the control group were born vaginally (51.7%) than in the abdominal surgery control group (42.9%) and the medically (36.5%) and surgically (33.9%) treated NEC groups. Approximately one in five babies with NEC were small for gestational age, compared with only 10.6% in the preterm control group, and 15.3% in those receiving other abdominal surgery. Babies with NEC also had lower Apgar scores, with respectively 32.3% and 38.8% of the medically and surgically treated groups, compared with 8.3% and 15.3% in the control group and abdominal surgery group. Parity and median maternal age were similar for all groups. A maternal history of any of the outcomes was recorded in the Birth Registry in 0.4–1.0% in the four groups. Only 0.3% of the infants received outpatient NSAIDs during the first year of life. Overall, 2.2% received PPIs and 14.9% systemic antibiotics, with the highest prevalence among those receiving abdominal surgery for indications other than NEC (14.6% and 26.3% respectively). Antibiotic use was similar in the three other groups, while PPI use was much higher in the surgical NEC group (13.3%) and medical NEC group (9.4%) than in the controls (1.9%).

### Neurodevelopmental outcomes

**Fig. 2:**
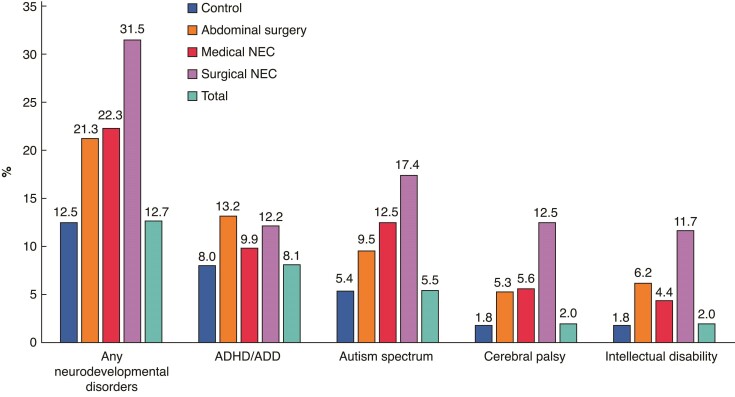
Prevalence of neurodevelopmental disorders for all preterm infants categorized as medically or surgically treated NEC, and abdominal surgery (1998–2019) ADHD/ADD, attention deficit (and hyperactivity) disorder; NEC, necrotizing enterocolitis.

**Fig. 3:**
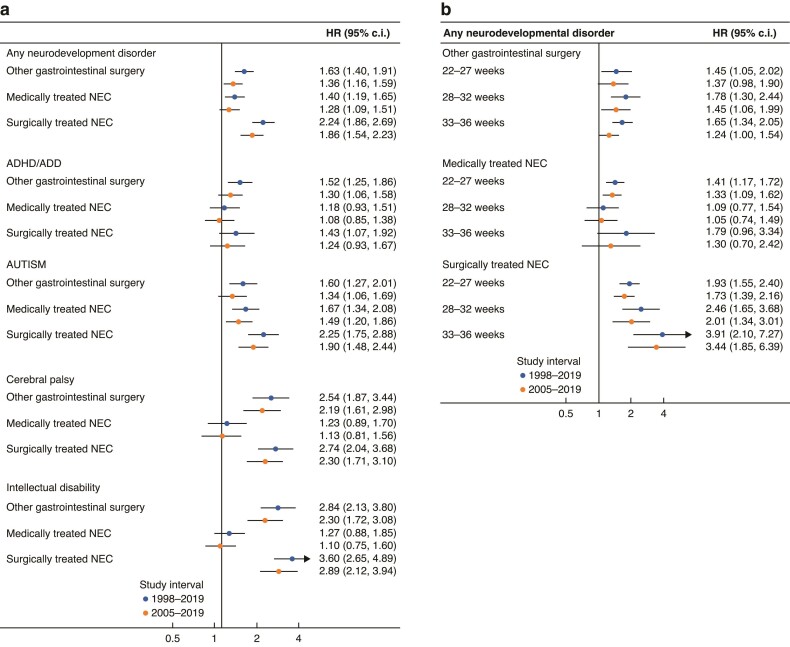
Association between medically and surgically treated necrotizing enterocolitis and abdominal surgery compared with all other preterm babies, expressed as hazard ratios (HR) and 95% confidence intervals (c.i.) **a** All different neurodevelopmental outcomes, **b** per gestational age. ADHD/ADD, attention deficit (and hyperactivity) disorder; NEC, necrotizing enterocolitis.

Overall, 12.7% of the total cohort developed at least one neurodevelopmental disorder, or approximately one-fifth of the abdominal surgery group and the medical NEC group, and one-third of the surgical NEC group (*Table 1* and *Fig. 2*). Overall (1998–2005), the adjusted HRs were highest for surgically treated NEC (HR 2.24, 95% c.i. 1.86 to 2.69) compared with the preterm control group, followed by the abdominal surgery group (HR 1.63, 95% c.i. 1.40 to 1.91) and the medically treated NEC group (HR 1.40, 95% c.i. 1.19 to 1.65) (*Fig. 3a*). After additional adjustments for early-life drug exposure (2005–2019), the results were similar, yet less extreme (*Fig. 3a*).

### ADHD/ADD

The median age of diagnosis was between 5.0 and 7.8 years (range 0–15 years), with the youngest age in the surgically treated NEC group, and the oldest age in the preterm control group (*Table 1*). ADHD/ADD was the most common neurodevelopmental disorder among the controls (8.0%) and the abdominal surgery (13.2%) groups (*Table 1* and *Fig. 2*). The prevalence in the surgical NEC group was, however, similar (12.2%) to the abdominal surgery group, and 9.9% of the medical NEC group was diagnosed (*Table 1* and *Fig. 2*). The adjusted HRs (1998–2019) were highest in the abdominal surgery group (HR 1.52, 95% c.i. 1.25 to 1.86), followed by the surgically treated NEC group (HR 1.43, 95% c.i. 1.07 to 1.92) compared with the preterm control group (*Fig. 3a*). After additional adjustment for early-life drug exposure (2005–2019), only the association between surgically treated NEC and ADHD/ADD remained significant (HR 1.30, 95% c.i. 1.06 to 1.58) (*Fig. 3a*).

### Autism spectrum

Median age of first diagnosis was between 3.6 and 5.3 years (range 0–15 years), with the youngest age in the medically treated NEC group, and the oldest age in the preterm control group (*Table 1*). Autism spectrum disorders were the most frequently recorded diagnosis in the medical (12.5%) and surgical NEC (17.4%) groups, compared with 5.4% and 9.4% in the preterm control group and the abdominal surgery controls (*Table 1* and *Fig. 2*). The adjusted HRs (1998–2005) were elevated in both NEC groups (surgical HR 2.25, 95% c.i. 1.75 to 2.88; medical HR 1.67, 95% c.i. 1.34 to 2.08) and the abdominal surgery control group (HR 1.60, 95% c.i. 1.27 to 2.01) compared with the preterm control group (*Fig. 3a*). After additional adjustments for prescribed drug use (2005–2019), all estimates remained significant, and in the same order, yet the effect sizes were less extreme (*Fig. 3a*).

### Cerebral palsy

The median age of the first recorded diagnosis was between 1.6 and 1.9 years (range 0–14.9 years), with the youngest age in the medically treated NEC group, and the oldest in the preterm control group (*Table 1*). The prevalence of cerebral palsy was highest among those receiving surgical treatment for NEC (12.5%), compared with medically treated NEC (5.6%), abdominal surgery controls (5.3%) and the preterm control group (1.8%) (*Table 1* and *Fig. 2*). The adjusted HRs (1998–2005) were most elevated for surgically treated NEC (HR 2.74, 95% c.i. 2.04 to 3.68) and the abdominal surgery control group (HR 2.54, 95% c.i. 1.87 to 3.44), compared with the preterm control group (*Fig. 3a*). The results were less pronounced after additional adjustments for early-life prescribed drug use (2005–2019, *Fig. 3a*). No statistically significant association was found between medically treated NEC and cerebral palsy.

### Intellectual disability

The median age of the first recorded diagnosis was 5.3–6.4 years in all four groups (range 0–14.9 years), with the youngest age in the surgically treated NEC group, and the oldest in the preterm control group (*Table 1*). The prevalence of intellectual disability was highest in the surgically treated NEC group (11.7%), followed by the abdominal surgery control group (6.2%), the medically treated NEC group (4.4%) and the control group (1.8%) (*Table 1* and *Fig. 2*). Compared with the preterm control group, the adjusted HRs were strongly elevated in both surgically treated groups compared with the preterm control group, with HR 3.60 (95% c.i. 2.65 to 4.90) and HR 2.84 (95% c.i. 2.13 to 3.80) in the surgical NEC group and abdominal surgery control groups respectively, and similar after the additional adjustments for early-life drugs (*Fig. 3a*). No association was found for medically treated NEC (*Fig. 3a*).

### Gestational age

Respectively 10.8%, 1.7% and 0.2% of all extremely preterm, early preterm and late preterm infants developed NEC (*Table 2*). Overall, 26.3% of the extremely preterm group, 15.6% of the early preterm and 10.1% of the late preterm group developed a neurodevelopmental disorder. The median age of diagnosis ranged between 3.6 years (extremely preterm) and 6.6 years (late preterm) (*Table 2*). For all neurodevelopmental disorders combined, the hazards in the abdominal surgery group appeared similar between the different gestational age groups (*Fig. 3b*). Yet, in the surgically treated NEC group, the effect seemed to increase by gestational age, with HR 3.44 (95% c.i. 1.85 to 6.39) for the late preterm group (33–36 weeks), compared with HR 1.73 (95% c.i. 1.39 to 2.16) for the extremely preterm group, yet with overlapping confidence intervals (*Fig. 3b*). In the medically treated NEC group, the association was only significant in the extremely preterm group (HR 1.33, 95% c.i. 1.09 to 1.62) (*Fig. 3b*). Among the specific outcomes per gestational age group (*Table S2*), the highest risk estimates were shown for autism spectrum disorders among the late preterm group with surgically treated NEC (HR 6.77, 95% c.i. 3.38 to 13.56), followed by the risk of intellectual disability in the early preterm group with surgical NEC (HR 5.14, 95% c.i. 2.64 to 9.99) and abdominal surgery (HR 4.56, 95% c.i. 2.80 to 7.42).

**Table 2:** Prevalence of neurodevelopmental disorders stratified by gestational age

Variable			
	22–27 weeks	28–32 weeks	33–36 weeks
Paediatric outcomes	*n* = 6194	*n* = 19 070	*n* = 54 957
Any neurodevelopmental outcome	1628 (26.3)	2982 (15.6)	5553 (10.1)
Age (years), median (range)	3.6 (0.1–15)	5.6 (0–15)	6.6 (0–15)
ADHD/ADD	821 (13.3)	1753 (9.2)	3878 (7.1)
Age (years), median (range)	6.6 (0.2–15)	7.9 (0–15)	7.9 (0–15)
Autism spectrum	824 (13.3)	1281 (6.7)	2319 (4.2)
Age (years), median (range)	4.3 (0.3–15)	5.2 (0–15)	5.5 (0.1–15)
Cerebral palsy	471 (7.6)	660 (3.5)	438 (0.8)
Age (years), median (range)	2 (0.2–14.7)	1.9 (0–14.9)	1.7 (0.1–14.1)
Intellectual disability	382 (6.2)	466 (2.4)	701 (1.3)
Age (years), median (range)	6.0 (0.1–14.7)	6.5 (0.4–14.9)	6.4 (0.5–14.9)
Death (first year)	946 (15.3)	400 (2.1)	318 (0.6)
**All NEC**	671 (10.8)	319 (1.7)	91 (0.2)
Medically treated	419 (6.8)	224 (1.2)	58 (0.1)
Surgically treated	252 (4.1)	95 (0.5)	33 (0.1)

Values are *n* (%) unless otherwise indicated. ADHD/ADD, attention deficit (and hyperactivity) disorder; NEC, necrotizing enterocolitis.

### Covariates

When looking at all neurodevelopmental disorders combined (1998–2019), all selected potential confounders reached statistical significance, with apparently protective factors including higher gestational age, female sex, Apgar ≥7 and a higher maternal age (per year increase). The largest risk factors included the presence of maternal neurodevelopmental disorder, small for gestational age, large for gestational age, elective or acute caesarian section and higher parity (*Fig. S1a*). Early-life exposure to PPIs (HR 3.08, 95% c.i. 2.81 to 3.38) seemed to have a stronger association than antibiotics (HR 1.25, 95% c.i. 1.19 to 1.31). When looking at the separate neurodevelopmental disorders, the results remained similar. For cerebral palsy, older gestational age seemed even more protective than for the other disorders, while maternal history of neurodevelopmental disorder appeared an important risk factor for intellectual disability (*Fig. S1b–e*). The highest risk estimates for each disorder separately (2005–2019) were found for early-life PPI exposure (HR 2.66 for ADHD/ADD, HR 3.09 for autism spectrum, HR 2.6 for cerebral palsy and HR 3.43 for intellectual disabilities) (*Fig. S1b–e*).

## Discussion

This large nationwide study suggests that surgically treated NEC, medically treated NEC and abdominal surgery for other indications in preterm infants were all associated with an increased risk of impaired neurodevelopmental outcomes, compared with other preterm infants. The results may be explained by the fact that the preterm brain seems to be extra sensitive to factors such as the systemic inflammation in NEC and general anaesthetic agents that may interrupt brain development^7,9,10,26^. The risk was most pronounced in the surgically treated group with NEC, after adjustments for multiple confounders and gestational age. The higher association between surgically treated NEC and risk of neurodevelopmental disorder could be partially explained by the higher severity of NEC, which triggered surgical intervention.

Brain injury in preterm infants with surgical NEC has been found to correlate with clinical and bowel pathological findings^27^. The results of this study are in agreement with a previous small study reporting significantly more brain injury determined using magnetic resonance imaging in six infants with surgically treated NEC compared with 25 infants with medically treated NEC, even after adjustments for confounders^28^. However, the increased risk estimates for all outcomes among those operated on for other abdominal indications suggest the procedure itself (including anaesthesia, antibiotics etc.) may also negatively affect the developing brain. A recent publication reported lower white matter volume at term equivalent age in 25 very preterm infants who required surgery during the preterm interval and lower cognitive and motor outcomes at 2 years^10^. Among those 25, three infants were surgically treated for NEC and two infants were medically treated^10^. For this project we only selected abdominal surgery as a comparison, as it is most similar to NEC surgery considering duration, type of anaesthesia, invasiveness and anatomical location, to reduce the effect of residual confounding and selection bias. Yet, this project suggests other types of neonatal surgery using general anaesthesia may also have negative effects on long-term brain development.

Interestingly, autism spectrum disorders were the only outcome reaching statistical significance among the medically treated group with NEC, which was also the most common neurodevelopmental disorder reported among those with surgically treated NEC. According to a systematic review, autism is already more prevalent in preterm infants than term infants^29^, but this study suggests the risk still varies depending on NEC and surgery. In the general preterm group and abdominal surgery groups, ADHD/ADD was most common. For surgically treated NEC, the risk was elevated in all gestational age groups and, counterintuitively, even more in the older infants than in the extremely preterm group. The hypothesis is that the different baseline risk (higher in the extremely preterm group than the late preterm) may mean that the additional harm of NEC and its treatment may be more pronounced in the older preterm infants. However, these results warrant further exploration, as it is difficult to differentiate the independent effects of the multiple risk factors.

In the abdominal surgery group, the effect did not change much among the different gestational age groups, while in the medical NEC group, the only effect was demonstrated in the extremely preterm group. These findings may suggest that the anaesthesia has a larger effect than the underlying systemic inflammation related to NEC on neurodevelopment.

The strengths of our study include the large sample size of almost 81 000 preterm infants, of whom more than 1000 developed NEC, with long follow-up (up to 19 years). In the current literature, there are only a few studies comparing neurodevelopmental outcomes in medical and surgical NEC, and the study groups are much smaller^10^. These nationwide population registries have been regarded as highly valid and unique resources for epidemiological research^18,30^. However, it is possible that ICD coding for prematurity (P07) may not have been applied to older preterm infants in the Swedish Medical Birth Registry, which was used by the National Board of Health and Welfare to select the infants for the cohort (*Fig. 1b*). The publicly available estimates from the National Board of Health and Welfare reported 141 326 preterm infants during the study interval, whilst this nationwide study only included 80 909 infants. All preterm children with NEC should have been recorded and those with the selected abdominal surgery codes (also used for identifying the cohort), and we may therefore have missed some children who should have been in the large preterm control group or were excluded because of chromosomal disorders. The 10.8% of NEC among the extremely preterm is in line with estimates of the meta-analysis of 27 studies (range 3–17%)^1^, which did not report specific estimates on early and late preterm. A validation study on the Swedish Registries describing an increased NEC incidence in Sweden concluded that NEC was often overdiagnosed, with suspected NEC (Bell Stage I) and spontaneous intestinal perforations (SIP) being misdiagnosed as NEC^31^. However, these results should be generalizable to other settings with well functioning, accessible neonatal and overall healthcare. No validation studies were identified on the Patient Registry specifically regarding neurodevelopmental outcomes, but the overall validity of this registry is considered high also for neuropsychiatric outcomes^32–34^.

In Sweden, antenatal care is highly standardized, accessible and free, and disparities in access to overall healthcare are relatively limited. High-risk infants who were admitted to neonatal care in Sweden are regularly followed up for 5.5 years to detect long-term functional impairments faster as these may be missed in routine child care^35^. This group includes, among others, all extremely preterm babies and children with morphological brain damage or central nervous system infections^35^. This implies the risk of underreporting and later diagnosis is lower for the extremely preterm infants than late preterm infants, which is also suggested by the lower age of diagnosis in the extremely preterm group. The time and probability of diagnosis should not differ depending on NEC history or surgery. However, children with persisting health issues may be more likely to be diagnosed because of more frequent contact with healthcare services. The open cohort design also implies that some individuals may have received a diagnosis after the end of follow-up and that some outcomes (particularly the less severe) may have been missed.

To account for differences in follow-up time in this open cohort, Cox proportional hazard modelling was used as all eligible children with a follow-up of at least 1 year could be included. To explore the data, multivariable logistic regression models were applied restricted to the subgroups with a full 5-year follow-up and 7-year follow-up, which yielded similar results and are therefore not presented. It is acknowledged that several of the children may have received an outcome diagnosis after follow-up, as some individuals will only be diagnosed during later childhood or even adulthood.

Although multiple potential confounders were adjusted, there may still be residual confounding. There was limited information on family history (only from mothers if reported in the medical birth registry), while paternal age and medical history may also affect the risk. Antibiotic intake during early life was also associated with increased risk of ADHD/ADD, autism spectrum disorders and intellectual disability, which could also be due to disruptions of the immature gut microbiome acquisition process. Yet, several (viral) infections have been linked with neurodevelopment, and with an increased risk of NEC^36^, and may (ineffectively) have been treated by antibiotics. It is more difficult to hypothesize how non-antibiotic drugs such as PPIs may affect the risk of impaired neurodevelopmental outcomes, if not through the gut microbiome as it seems unlikely acid-related disorders would affect brain development. Yet, PPIs might be prescribed for vague indications, for example suboptimal sleeping patterns presumed to be related to gastric acidity/reflux, whilst these could already be an early sign of neurodevelopmental problems. Further exploration of early-life exposure to non-antibiotic drugs on neurodevelopment and other outcomes, as well as the microbiome, seems warranted as also suggested by a recent study linking antenatal antipsychotics to various neonatal outcomes^37^. Other neurodevelopmental and psychiatric outcomes including intellectual functioning, substance use and mood and psychotic disorders may also need further exploration, as the risk appears to be higher after preterm birth compared with term birth^38,39^.

The implications of our study are mainly aetiological and may help understand the complex pathophysiology of NEC and neurodevelopment, and hopefully also contribute somehow to better prevention and earlier detection and support of neurodevelopmental disorders^8^. Preterm birth is described as the most important risk factor for impaired neurodevelopmental outcomes during childhood, with a worldwide impact^12,40^. Several mechanisms have been proposed linking prematurity and NEC to impaired neurodevelopmental outcomes including through the gut–brain microbiome axis and epigenetic changes^8,40–49^. Surgery may also have direct or indirect neurological effects due to anaesthesia or other drugs, poor perioperative oxygenation, the perioperative pain and/or inflammatory processes^26^. It appears anaesthesia and perioperative interventions and complications pose the highest risk for the youngest (most immature) and oldest brains, as the harmful effects of anaesthesia and surgery are well known and intensively investigated among the elderly^41–45^.

The study findings may have clinical implications as they might help in decision-making regarding different medical or surgical interventions, as surgery may be life-saving but the benefit has been questioned if no bowel perforation is present^50^. Although survival remains a major concern in NEC, before longer-term neurodevelopment^51–53^, it is important to discuss neuroprotection practices during surgery in preterm infants^54^. Timely NEC diagnosis and initiation of appropriate antimicrobial treatment are crucial, and delayed antimicrobial treatment is associated with a higher need for surgery^55–57^. The potential overtreatment of suspected, non-confirmed sepsis and NEC has been acknowledged^58,59^. Longer term and neurodevelopmental outcomes have not yet been addressed when comparing different surgical approaches for NEC^50,60–63^. No strong conclusions can be made about individual ‘causes’ of neurodevelopmental disorders as there is clearly a multifactorial origin, especially since this is an extremely vulnerable population with a high risk of mortality and morbidity^63^. Survival has improved for preterm infants, even for extremely preterm infants, but many still have life-long consequences which makes it worthwhile exploring if there are possibilities to reduce these risks. In absolute numbers, the risk of autism spectrum disorders may be particularly affected by NEC, as it is the most frequent neurodevelopmental disorder in both NEC groups. The risks of cerebral palsy and intellectual disability were most increased compared with the preterm control group.

## Supplementary Material

zrae131_Supplementary_Data

## Data Availability

The data sets generated and analysed during the present study are not publicly available due to restrictions from the National Board of Health and Welfare (Socialstyrelsen), which owns the data. The data are available from the corresponding author on reasonable request after required approvals from the national Ethics Committee and National Board of Health and Welfare.
