# Supplementary material for: Risk of neurodevelopmental impairment in Swedish preterm children treated for necrotizing enterocolitis: retrospective cohort study
Source: BJS Open. 2024 Nov 8;8(6):zrae131. doi: 10.1093/bjsopen/zrae131 (PMC11544313; doi:10.1093/bjsopen/zrae131)
Supplement: zrae131_Supplementary_Data [file zrae131_supplementary_data.docx]

**The risk of neurodevelopmental impairment in Swedish preterm children treated for necrotizing enterocolitis: Retrospective cohort study**

Nele BRUSSELAERS,^1,2,3^, Johanna SIMIN^2^, Helene E. LILJA^1,4^

^1^Department of Women's and Children's Health, Karolinska Institutet, Stockholm, Sweden

^2^Global Health Institute, Department of Family Medicine and Population Health, University of Antwerp, Antwerp, Belgium

^3^Department of Public Health and Primary Care, Ghent University, Ghent, Belgium

^4^Department of Paediatric Surgery, Karolinska University Hospital, Solna, Stockholm, Sweden

**Corresponding author.** Nele Brusselaers, [nele.brusselaers@ki.se](mailto:nele.brusselaers@ki.se); https://orcid.org/0000-0003-0137-447X

**Supplementary Materials - Index**

| **Supplementary Figures and Tables** |  |
| --- | --- |
| ***Supplementary table 1***: Codes and study periods of all variables used. | *page 2-3* |
| ***Supplementary table 2***: Association between surgical and medically treated NEC, and abdominal surgery compared to all other preterm babies, expressed as hazard ratios (HR) and 95% confidence intervals (CI), by time period, and by gestational age. | *page 4* |
| ***Supplementary Figures 1a-e***: association of all covariates and the risk of (a) any neurodevelopmental outcome, (b) attention deficit (and hyperactivity) disorders (ADHD/ADD), (c) autism spectrum disorders, (d) cerebral palsy, and (e) intellectual disability, expressed as multivariable hazard ratios (HR) and 95% confidence intervals. | *Page 5-6* |
|  |  |
|  |  |
|  |  |

**Supplementary Figures and Tables**

**Supplementary table 1: Codes and study periods of all variables used.**

| **Code**  **International Classification of Diseases (ICD)- 10th edition** | **Variable** | **Registry** | **Time period** |
| --- | --- | --- | --- |
| ***Inclusion criteria*** |  |  |  |
| P77 | Necrotizing enterocolitis (intestinal inflammation with tissue death in fetuses and newborns) | In- and outpatient registry | 1998-2019 |
| P07 | Preterm birth (Disorders associated with prematurity and low birth weight not classified elsewhere | In- and outpatient registry | 1998-2019 |
| ***Exclusion criteria*** |  |  |  |
| Q90-99 | Chromosomal disorders | In- and outpatient registry | 1998-2019 |
|  |  |  |  |
| ***Intra-abdominal surgery - Nordic Medico-Statistical Committee (NOMESCO) Classification of Surgical Procedures*** | |  |  |
| JF | Intestine surgery | In-patient registry | 1998-2020 |
| >JFB | Partial excision of intestine (NEC surgery) | In-patient registry | 1998-2020 |
| >JFF | Exteriorization of intestine and creation of intestinal stomas (NEC surgery) | In-patient registry | 1998-2020 |
| JAM10 | Operation for malrotation of intestine | In-patient registry | 1998-2020 |
| JAG20 | Repair of omphalocele | In-patient registry | 1998-2020 |
| JAG30 | Reconstruction of abdominal wall using flap | In-patient registry | 1998-2020 |
| JAG60 | Reconstruction of abdominal wall using prosthetic material | In-patient registry | 1998-2020 |
| JDH40 | Duodenostomy on duodenal bulb | In-patient registry | 1998-2020 |
| ***Outcomes - ICD 10*** |  |  |  |
| F70-79 | Intellectual disability (Psychological development disorders) | In- and outpatient registry | 1998-2020 |
| F84 | Autism spectrum | In- and outpatient registry | 1998-2020 |
| F90 | ADHD/ADD | In- and outpatient registry | 1998-2020 |
| G80 | Cerebral palsy | In- and outpatient registry | 1998-2020 |
| ***Censoring based on death*** |  |  |  |
| Any cause of death | Any cause of death | Death Registry | 1998-2020 |
| ***Potential confounders*** |  |  |  |
| KON | Sex of child | Medical Birth Registry | 1998-2019 |
| GRVFV | Gestational length | Medical Birth Registry | 1998-2019 |
| SECTIO, SECAFSL, SECFORE, SECMARK | Mode of delivery | Medical Birth Registry | 1998-2019 |
| APGAR5 | APGAR score 5 minutes | Medical Birth Registry | 1998-2019 |
| PARITET | Parity | Medical Birth Registry | 1998-2019 |
| MSGA, MLGA | Birth weight by gestational age (Small for gestational age, Large for gestational age) | Medical Birth Registry | 1998-2019 |
| MDIAGNOS, MDIAG1-12 | Maternal diagnosis (Autism spectrum, ADHD/ADD, Intellectual disability, Cerebral palsy - F70-79, F84, F90, G80 ) | Medical Birth Registry | 1998-2019 |
| MALDER | Maternal age | Medical Birth Registry | 1998-2019 |
| ***Prescribed outpatient drug use - Anatomical Therapeutic Chemical (ATC) classification during first year of life*** | |  |  |
| A02BC | Proton pump inhibitors | Prescribed Drug Registry | July 2005-2020 |
| J01, P01AB | Systemic antibiotics | Prescribed Drug Registry | July 2005-2020 |
| M01 | Non-steroidal anti-inflammatory drugs | Prescribed Drug Registry | July 2005-2020 |

**Supplementary table 2: Association between surgical and medically treated NEC, and abdominal surgery compared to all other preterm babies, expressed as hazard ratios (HR) and 95% confidence intervals (CI), by time period, and by gestational age.**

| **Total cohort** | **Any neurodevelopmental outcome** | | **ADHD/ADD** | | **Autism-spectrum** | | **Cerebral palsy** | | **Intellectual disability** | |
| --- | --- | --- | --- | --- | --- | --- | --- | --- | --- | --- |
| ***1998-2005*** | ***HR*** | ***(95% CI)*** | ***HR*** | ***(95% CI)*** | ***HR*** | ***(95% CI)*** | ***HR*** | ***(95% CI)*** | ***HR*** | ***(95% CI)*** |
| Other intra-abdominal surgery | **1.63** | **(1.40-1.91)** | **1.52** | **(1.25-1.86)** | **1.60** | **(1.27-2.01)** | **2.54** | **(1.87-3.44)** | **2.84** | **(2.13-3.80)** |
| Medically treated NEC | **1.40** | **(1.19-1.65)** | 1.18 | (0.93-1.51) | **1.67** | **(1.34-2.08)** | 1.23 | (0.89-1.70) | 1.27 | (0.88-1.85) |
| Surgically treated NEC | **2.24** | **(1.86-2.69)** | **1.43** | **(1.07-1.92)** | **2.25** | **(1.75-2.88)** | **2.74** | **(2.04-3.68)** | **3.60** | **(2.65-4.89)** |
| ***2005-2019*** |  |  |  |  |  |  |  |  |  |  |
| Other intra-abdominal surgery | **1.36** | **(1.16-1.59)** | **1.30** | **(1.06-1-58)** | **1.34** | **(1.06-1.69)** | **2.19** | **(1.61-2.98)** | **2.30** | **(1.72-3.08)** |
| Medically treated NEC | **1.28** | **(1.09-1.51)** | 1.08 | (0.85-1.38) | **1.49** | **(1.20-1.86)** | 1.13 | (0.81-1.56) | 1.10 | (0.75-1.60) |
| Surgically treated NEC | **1.86** | **(1.54-2.23)** | 1.24 | (0.93-1.67) | **1.90** | **(1.48-2.44)** | **2.30** | **(1.71-3.10)** | **2.89** | **(2.12-3.94)** |
|  |  |  |  |  |  |  |  |  |  |  |
|  |  |  |  |  |  |  |  |  |  |  |
| ***1998-2005*** | **Any neurodevelopmental outcome** | | **ADHD/ADD** | | **Autism-spectrum** | | **Cerebral palsy** | | **Intellectual disability** | |
| 22-27 wks | ***HR*** | ***(95% CI)*** | ***HR*** | ***(95% CI)*** | ***HR*** | ***(95% CI)*** | ***HR*** | ***(95% CI)*** | ***HR*** | ***(95% CI)*** |
| Other intra-abdominal surgery | **1.45** | **(1.05-2.02)** | **1.97** | **(1.31-2.97)** | 1.42 | (0.89-2.27) | **1.96** | **(1.15-3.35)** | 1.22 | (0.58-2.58) |
| Medically treated NEC | **1.41** | **(1.17-1.72)** | 1.16 | (0.85-1.56) | **1.63** | **(1.26-2.11)** | 1.24 | (0.86-1.81) | 1.31 | (0.86-2.01) |
| Surgically treated NEC | **1.93** | **(1.55-2.40)** | 1.17 | (0.81-1.70) | **1.96** | **(1.46-2.64)** | **2.76** | **(1.99-3.83)** | **3.13** | **(2.19-4.47)** |
| 28-32 wks |  |  |  |  |  |  |  |  |  |  |
| Other intra-abdominal surgery | **1.78** | **(1.30-2.44)** | **1.56** | **(1.03-2.38)** | **1.80** | **(1.14-2.83)** | **2.74** | **(1.61-4.66)** | **4.56** | **(2.80-7.42)** |
| Medically treated NEC | 1.09 | (0.77-1.54) | 1.05 | (0.65-1.69) | 1.52 | (0.96-2.39) | 1.05 | (0.50-2.21) | 1.30 | (0.58-2.91) |
| Surgically treated NEC | **2.46** | **(1.65-3.68)** | **1.91** | **(1.05-3.46)** | **2.11** | **(1.13-3.94)** | **3.01** | **(1.50-6.07)** | **5.14** | **(2.64-9.99)** |
| 33-36 wks |  |  |  |  |  |  |  |  |  |  |
| Other intra-abdominal surgery | **1.65** | **(1.34-2.05)** | **1.35** | **(1.03-1.77)** | **1.68** | **(1.21-2.32)** | n.a. | n.a. | n.a. | n.a. |
| Medically treated NEC | 1.79 | (0.96-3.34) | 1.58 | (0.71-3.53) | 2.12 | (0.88-5.09) | n.a. | n.a. | n.a. | n.a. |
| Surgically treated NEC | **3.91** | **(2.10-7.27)** | **2.92** | **(1.31-6.51)** | **6.77** | **(3.38-13.56)** | n.a. | n.a. | n.a. | n.a. |
| **2005-2019** |  |  |  |  |  |  |  |  |  |  |
| 22-27 wks |  |  |  |  |  |  |  |  |  |  |
| Other intra-abdominal surgery | 1.37 | (0.98-1.90) | **1.90** | **(1.26-2.86)** | 1.35 | (0.84-2.16) | **1.88** | **(1.10-3.21)** | 1.16 | (0.55-2.46) |
| Medically treated NEC | **1.33** | **(1.09-1.62)** | 1.09 | (0.80-1.47) | **1.50** | **(1.15-1.94)** | 1.18 | (0.81-1.72) | 1.23 | (0.80-1.88) |
| Surgically treated NEC | **1.73** | **(1.39-2.16)** | 1.09 | (0.75-1.58) | **1.76** | **(1.31-2.37)** | **2.51** | **(1.80-3.51)** | **2.85** | **(1.98-4.10)** |
| 28-32 wks |  |  |  |  |  |  |  |  |  |  |
| Other intra-abdominal surgery | **1.45** | **(1.06-1.99)** | 1.26 | (0.82-1.93) | 1.47 | (0.93-2.32) | **2.25** | **(1.31-3.85)** | **3.32** | **(2.01-5.48)** |
| Medically treated NEC | 1.05 | (0.74-1.49) | 1.03 | (0.64-1.66) | 1.48 | (0.94-2.33) | 1.01 | (0.48-2.14) | 1.28 | (0.57-2.87) |
| Surgically treated NEC | **2.01** | **(1.34-3.01)** | 1.60 | (0.88-2.91) | 1.72 | (0.92-3.22) | **2.59** | **(1.28-5.22)** | **4.00** | **(2.05-7.83)** |
| 33-36 wks |  |  |  |  |  |  |  |  |  |  |
| Other intra-abdominal surgery | **1.24** | **(1.00-1.54)** | 1.10 | (0.84-1.45) | 1.24 | (0.89-1.73) | n.a. | n.a. | n.a. | n.a. |
| Medically treated NEC | 1.30 | (0.70-2.42) | 1.21 | (0.54-2.71) | 1.51 | (0.63-3.65) | n.a. | n.a. | n.a. | n.a. |
| Surgically treated NEC | **3.44** | **(1.85-6.39)** | **2.66** | **(1.20-5.94)** | **4.99** | **(2.48-10.04)** | n.a. | n.a. | n.a. | n.a. |

*ADHD/ADD, attention deficit (and hyperactivity) disorder; NEC, necrotizing enterocolitis.*

**Supplementary Figures 1a-e: association of all covariates and the risk of (a) any neurodevelopmental outcome, (b) attention deficit (and hyperactivity) disorders (ADHD/ADD), (c) autism spectrum disorders, (d) cerebral palsy, and (e) intellectual disability, expressed as multivariable hazard ratios (HR) and 95% confidence intervals.**


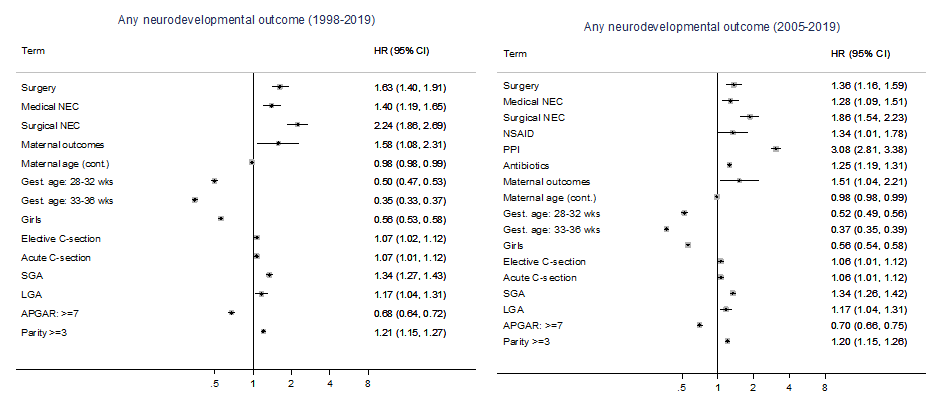


*1a*


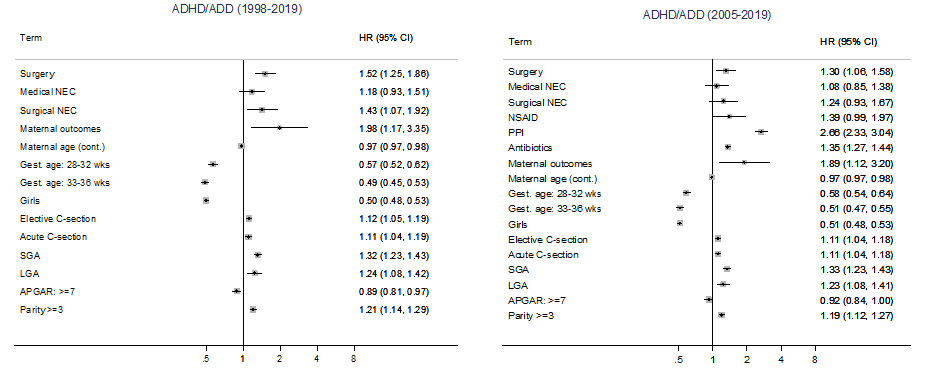
*1b*


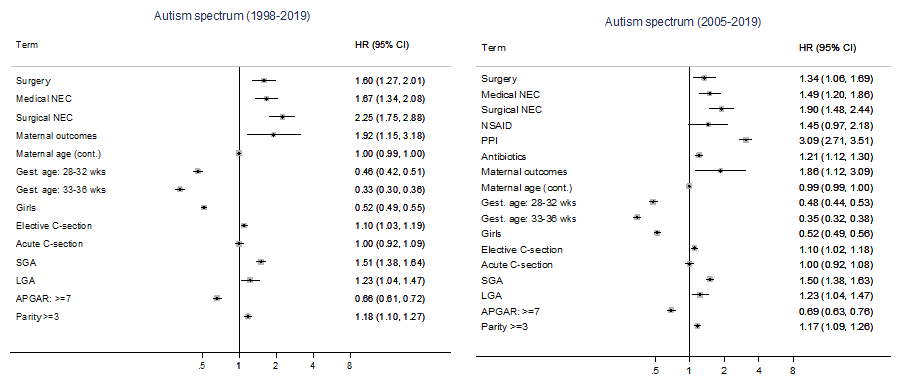
*1c*


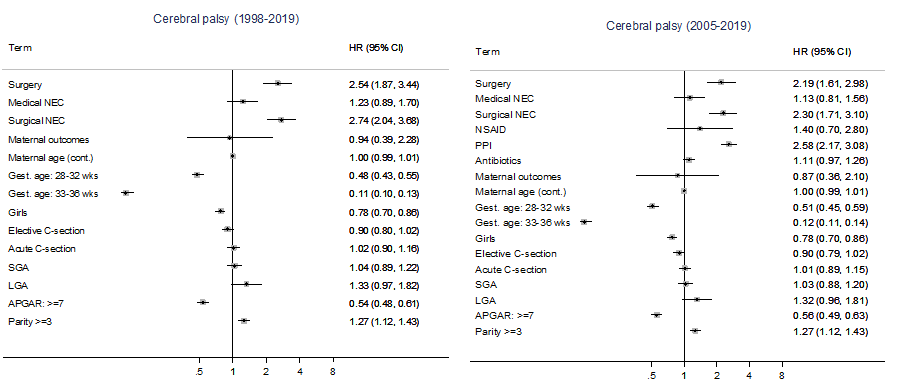
*1d*


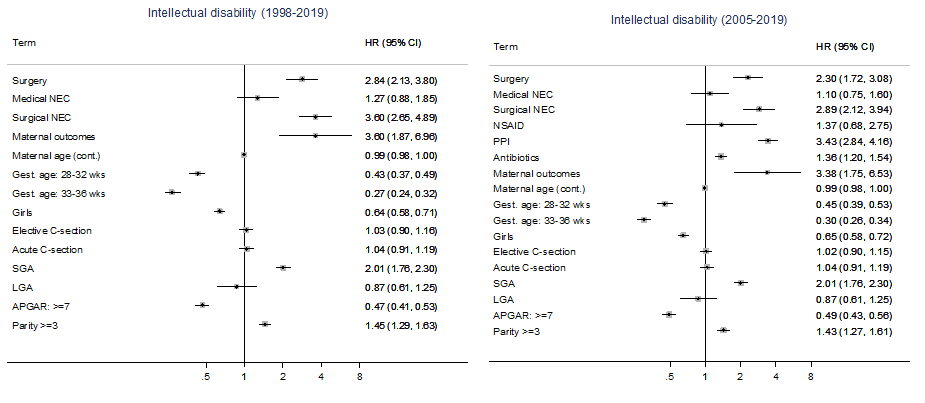
*1e*

*ADHD/ADD, attention deficit (and hyperactivity) disorder; NEC, necrotizing enterocolitis*; *NSAIDS, non-steroidal anti-inflammatory drugs; PPI, proton pump inhibitors.*
